# Neural Speech Tracking in the Theta and in the Delta Frequency Band Differentially Encode Clarity and Comprehension of Speech in Noise

**DOI:** 10.1523/JNEUROSCI.1828-18.2019

**Published:** 2019-07-17

**Authors:** Octave Etard, Tobias Reichenbach

**Affiliations:** Department of Bioengineering and Centre for Neurotechnology, Imperial College London, South Kensington Campus, SW7 2AZ, London, United Kingdom

**Keywords:** envelope tracking, neural oscillations, speech comprehension, speech processing

## Abstract

Humans excel at understanding speech even in adverse conditions such as background noise. Speech processing may be aided by cortical activity in the delta and theta frequency bands, which have been found to track the speech envelope. However, the rhythm of non-speech sounds is tracked by cortical activity as well. It therefore remains unclear which aspects of neural speech tracking represent the processing of acoustic features, related to the clarity of speech, and which aspects reflect higher-level linguistic processing related to speech comprehension. Here we disambiguate the roles of cortical tracking for speech clarity and comprehension through recording EEG responses to native and foreign language in different levels of background noise, for which clarity and comprehension vary independently. We then use a both a decoding and an encoding approach to relate clarity and comprehension to the neural responses. We find that cortical tracking in the theta frequency band is mainly correlated to clarity, whereas the delta band contributes most to speech comprehension. Moreover, we uncover an early neural component in the delta band that informs on comprehension and that may reflect a predictive mechanism for language processing. Our results disentangle the functional contributions of cortical speech tracking in the delta and theta bands to speech processing. They also show that both speech clarity and comprehension can be accurately decoded from relatively short segments of EEG recordings, which may have applications in future mind-controlled auditory prosthesis.

**SIGNIFICANCE STATEMENT** Speech is a highly complex signal whose processing requires analysis from lower-level acoustic features to higher-level linguistic information. Recent work has shown that neural activity in the delta and theta frequency bands track the rhythm of speech, but the role of this tracking for speech processing remains unclear. Here we disentangle the roles of cortical entrainment in different frequency bands and at different temporal lags for speech clarity, reflecting the acoustics of the signal, and speech comprehension, related to linguistic processing. We show that cortical speech tracking in the theta frequency band encodes mostly speech clarity, and thus acoustic aspects of the signal, whereas speech tracking in the delta band encodes the higher-level speech comprehension.

## Introduction

Speech comprehension requires real-time extraction of acoustic features from sound signals and their transformation into linguistic representations such as syllables, words, and phrases. The time scales of these linguistic structures match those of neural activities in the cerebral cortex, namely in the theta frequency band (4–8 Hz) that is comparable to the rate of syllables, and in the delta frequency band (1–4 Hz) that contains the time scale of words and phrases. The cortical activity in the delta and theta frequency band has indeed been found to track the speech rhythm, evident in its envelope, and this entrainment has been suggested as a neural mechanism for parsing speech into linguistic constituents (Ding and Simon, 2012, 2014; Giraud and Poeppel, 2012; Horton et al., 2013; Thwaites et al., 2015; Keitel et al., 2018). Evidence for this hypothesis comes from the modulation of cortical entrainment to the speech envelope through attention to one of several competing speakers, and cortical tracking may be particularly important for understanding speech in adverse conditions such as background noise (Ding and Simon, 2012; Horton et al., 2013; O'Sullivan et al., 2015).

Whether cortical speech tracking is involved in higher-level linguistic processing, beyond lower-level acoustic processing such as onset detection, remains, however, debated. Whereas some studies have found that cortical entrainment is stronger when speech is comprehended (Ahissar et al., 2001; Peelle et al., 2013; Ding et al., 2014; Pérez et al., 2015; Mai et al., 2016; Vanthornhout et al., 2018), others found no difference in the cortical tracking of intelligible and unintelligible speech sounds (Howard and Poeppel, 2010; Peña and Melloni, 2012; Millman et al., 2015; Mai et al., 2016; Zoefel and VanRullen, 2016). A main difficulty in assessing the role of cortical tracking for speech processing is that a difference of speech comprehension typically arises from a change in the acoustics, such as through a different level of background noise or through speech vocoding. This makes it difficult to tease apart which changes in cortical responses are because of lower-level acoustic alterations and which emerge from high-level linguistic processing. Cortical entrainment is indeed not unique to speech but has been observed in response to rhythmic non-speech stimuli such as reverse speech (Howard and Poeppel, 2010), music (Doelling and Poeppel, 2015; Meltzer et al., 2015), or frequency-modulated tones (Henry and Obleser, 2012).

**Figure 1.**
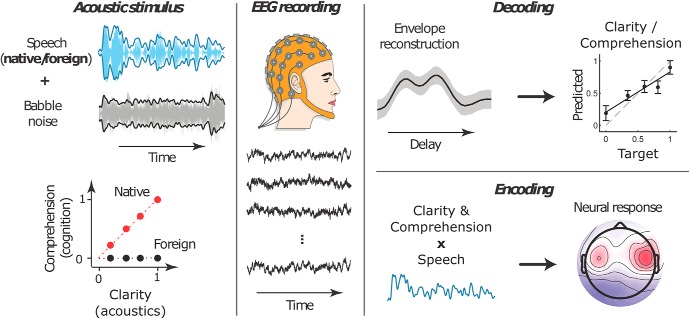
Overview of the experiment and analysis. Speech signals in both the subjects' native language English as well as in the foreign language Dutch were presented in different levels of background noise. We obtained both behavioral responses of the resulting speech clarity and comprehension as well as EEG recordings. EEG correlates of speech clarity and comprehension were then identified through both a decoding and an encoding approach. The decoding estimated clarity and comprehension from the cortical tracking of the speech envelope. The encoding approach reconstructed the EEG data from the speech envelope weighted by clarity and comprehension.

Here we used an experimental paradigm that enabled us to differentiate speech clarity from speech comprehension, and to accordingly segregate the roles of cortical entrainment in each of these two aspects of speech processing. Speech clarity is hereby considered as a function of the acoustic signal only: it measures how well a speech sound in the presence of certain degradations such as reverberation or background noise can be understood by a native speaker (Tasko and Greilick, 2010; Brezina, 2015; Signoret et al., 2018). Speech comprehension, in contrast, assesses how well a person has in fact understood a certain speech signal (Hickok and Poeppel, 2007; Price, 2012). We varied both factors independently by presenting native English speakers with speech in their native language in different levels of background noise, as well as with the foreign language Dutch in the same levels of background noise (Figure 1).

English and Dutch are both stress-timed languages and have comparable acoustics (Ramus et al., 1999). The English and the Dutch audio material were acoustically matched and thus had the same clarity that varied systematically from high to low with increasing background noise. The participants' speech comprehension, however, agreed with clarity only for the English speech but was nil for the Dutch. This experimental design allowed us to disambiguate the roles of cortical tracking for speech clarity, reflecting the lower-level acoustic processing, and speech comprehension, representing higher-level linguistic aspects.

## Materials and Methods

### 

#### 

##### Experimental design and statistical analysis.

We first assessed the comprehension of spoken English in varying levels of background noise behaviorally. We used the results to define four levels of background noise; that is, no, low, medium, and high background noise (Table 1). The levels were chosen so that speech comprehension varied from high to low with increasing noise.

**Table 1.** Experimental design: acoustic stimuli during neural data acquisition

Language	Background	SNR	Clarity, %	Comprehension, %
English	Quiet	N.A.	100	100
	Low babble noise	0.4	81	81
	Medium babble noise	−1.4	60	60
	High babble noise	−3.2	34	34
Dutch	Quiet	N.A.	100	0
	Low babble noise	0.4	81	0
	Medium babble noise	−1.4	60	0
	High babble noise	−0.32	34	0

We then presented continuous natural speech in both English and Dutch at the four different levels of background noise while recording EEG (Fig. 1). In particular, we used no noise such that speech was presented in a quiet condition, and we also used low, medium and high babble noise as defined above (Table 1). Speech comprehension decreased from 100% to 81, 60, and 34% from English without background noise to English presented in low, medium, and high babble noise. For Dutch, speech comprehension was 0% for all different noise levels: subjects did not understand the Dutch audio material beyond very few single and isolated words. However, we verified that subjects nonetheless attended the Dutch audio stimuli during the EEG recordings. In particular, subjects had to answer questions about rare English sentences that were embedded in the Dutch speech material as detailed below.

We defined the clarity of speech presented in a certain level of background noise, either for English or for Dutch, to be equal to the level of speech comprehension for the English stimuli in that noise level. Speech clarity was therefore 100, 81, 60, and 34% for either English or Dutch presented in no noise and low, medium and high babble noise, respectively (Table 1). Because speech clarity thereby was non-vanishing and varying for the Dutch stimuli, but speech comprehension for them was nil, the two variables speech clarity and speech comprehension varied independently. Our subsequent analysis sought to relate the four different levels of speech clarity as well as well as the five different levels of speech comprehension to the neural data, and thereby to disambiguate neural correlates of purely acoustic processing from those of speech comprehension.

To analyze the data we used both a decoding and an encoding approach of speech clarity and comprehension (Fig. 1). The neural response to the speech envelope occurs in the low-frequency range, 1–12 Hz, encompassing the delta, theta, and alpha frequency bands. The neural response in these different frequency bands may be affected by a change in the lower-level acoustical features, by higher-level speech comprehension, or by both. To disentangle neural correlates of speech comprehension from those of speech clarity we used two independent analyses. First, in a decoding approach, we determined the cortical tracking of the speech envelope. We then decoded both speech clarity and comprehension from the shape and magnitude of the envelope response. Second, we used an encoding approach in which we estimated the EEG recordings through the speech envelope weighted by speech clarity as well as through the envelope weighted by comprehension.

##### Participants.

Ten volunteers (aged 22.5 ± 0.6 year, all male) participated in the psychoacoustics part of the study. Twelve volunteers (aged 22.9 ± 2.5 year, 4 females) took part in the EEG recordings, three of which had undergone the behavioral testing as well. All participants were English native speakers with no history of auditory or neurological impairments, were right-handed, and provided written informed consent. The experimental procedures were approved by the Imperial College Research Ethics Committee.

##### Psychoacoustics.

We used a modified version of the Quick Speech-In-Noise test to measure speech comprehension in babble noise (Killion et al., 2004). Briefly, subjects were presented diotically with 72 English sentences in babble noise, and were asked to repeat them. An experimenter graded their responses based on five keywords. An adaptive Bayesian procedure was used to fit a sigmoidal psycho-acoustic curve *c*(*x*) = [1 + *e*^−β(*x*−α)^]^−1^, where *c* is the comprehension, *x* denotes the SNR, and α and β are two parameters to determine (Shen and Richards, 2012; Shen et al., 2015). The psychoacoustic curves were averaged over subjects, and the mean was used to determine comprehension level at a given SNR.

##### Acoustic stimuli for EEG recordings.

Non-repeating English and Dutch speech-in-noise stimuli were constructed by combining a target female speaker narrating continuous stories with four-speaker babble noise. The audio material for the target voice was obtained from publicly available audiobooks (http://librivox.org). The English babble noise was obtained from Auditec, and the Dutch babble noise was constructed by normalizing two female and two male voices to the same root-mean-square value and adding them. The target stories and background noise were then combined at three SNRs (0.4, −1.4, −3.2 dB). Additionally, one stimulus in English as well as another in Dutch with no background noise was produced. We thus obtained eight different acoustic stimuli (speech in 2 languages, each embedded in four noise levels). Each stimulus lasted ∼10 min, and was divided in four parts of similar duration. In the Dutch stimuli, a pseudorandom number of English sentences, between one and three, spoken by the same speaker as the target Dutch voice were inserted in each part, for a total of eight in each Dutch condition.

Both the English and the Dutch speech stimuli were different for each level of background noise, so that no subject heard the same speech twice. We therefore avoided priming effects that can result from repetitions (Rugg, 1985).

##### Neural data acquisition and preprocessing.

The English and Dutch stimuli were presented diotically in two sessions during which EEG was recorded. In each session, the stimuli were presented in order of decreasing SNR to maximize engagement, and the subject was allowed to rest for as long as they desired between each part. To ensure the subjects were attending to the stimuli, comprehension questions were asked after each part during the English session. During the Dutch session, the volunteers were instructed to listen to the story as if it were English, and were asked to identify, out of four choices, which English sentences had appeared in the excerpt they had just heard.

Scalp EEG was recorded at 1 kHz with 64 active electrodes positioned according to the standard 10–20 system actiCAP (actiCHamp, Brain Products), and referenced to the right earlobe. The audio stimuli were simultaneously recorded at 1 kHz by the amplifier through an acoustic adapter (Acoustical Stimulator Adapter and StimTrak, Brain Products), and this channel was used to temporally align the EEG data and the stimuli. The EEG data from one participant was too noisy to be used, and discarded. In the Dutch conditions, the data corresponding to the embedded English sentences was excluded from further analysis.

The raw EEG recordings were first detrended using routines from the Noise Toolbox (de Cheveigné and Arzounian, 2018). This involves fitting and subtracting a low-order polynomial to each recording part to remove slow drifts. The data were then low-pass filtered <12 Hz [linear phase FIR filter, cutoff (−6 dB) 12.5 Hz; transition bandwidth 1 Hz, order 3204, one-pass forward and compensated for delay], downsampled to 100 Hz, and high-pass filtered >1 Hz [linear phase FIR filter, cutoff (−6 dB) 0.5 Hz, order 322; transition bandwidth 1 Hz, one-pass forward and compensated for delay] using functions from the EEGLAB toolbox (Delorme and Makeig, 2004). Bad and missing channels were interpolated, and the recordings were re-referenced to the average, resulting in 1.6 ± 0.5 interpolated channels on average per subject. Infomax independent component analysis (ICA) as implemented in the AMICA algorithm was then run on the data for each subject and condition (Palmer et al., 2012). The obtained sets of independent components were conservatively pruned to remove large artifactual components such as eye movements.

For the decoding approach (backward models), the original detrended recordings were then bandpass filtered in the delta range (1–4 Hz), theta range (4–8 Hz) and alpha range (8–12 Hz) [high-pass: linear phase FIR filters, cutoff (−6 dB): 0.5 Hz (delta), 4 Hz (theta), 8 Hz (alpha), order: 162; low-pass: linear phase FIR filters, cutoff (−6 dB): 4 Hz (delta), 8 Hz (theta), 12.5 Hz (alpha), order 3204; all: transition bandwidth 1 Hz, one-pass forward and compensated for delay], and downsampled to 50 Hz. The cleaning parameters and ICA weights previously determined on the broadband (1–12 Hz) recordings were then applied to the corresponding datasets, hence ensuring that the same preprocessing was applied to all three bands.

For the encoding approach (forward model), the original detrended recordings were processed similarly. They were filtered in either the delta, theta or alpha bands [low-pass: linear phase FIR filters, cutoff (−6 dB): 4 Hz (delta), 8 Hz (theta), 12.5 Hz (alpha), order: 3204; high-pass: linear phase FIR filters, cutoff (−6 dB): 4 Hz (theta), 8 Hz (alpha), order 322; all: transition bandwidth 1 Hz, one-pass forward and compensated for delay], and downsampled to 100 Hz. The cleaning parameters and ICA weights previously determined on the broadband (1–12 Hz) recordings were then applied to the corresponding datasets.

These filters were noncausal filters that are designed to preserve the timing of the output but that violate causality by invoking future inputs in the computation of the output. To investigate potential artifacts of the noncausal filtering, we also filtered the recordings using a causal minimum-phase low-pass filter [FIR filter, cutoff (−6 dB) 16 Hz, order 202, transition bandwidth 16 Hz]. These causal filters were constructed such that the output depends only on the past and on the present of the input, and chosen to minimize the introduced delay at the expense of additional distortions (Press et al., 1986).

##### Computation of the speech envelope.

To compute the envelopes of the target speakers we first ran the speech signals through a low-pass filter imitating the earphones' frequency response [linear phase FIR filter, cutoff (−6 dB) 3500 Hz, transition bandwidth 1000 Hz, one-pass forward and compensated for delay], downsampled them from 44,100 to 8000 Hz and applied a full-wave rectification. The auditory periphery uses a nonlinear compression of the amplitude of a sound, and the cortical tracking of the speech envelope emerges particularly strongly when the nonlinear compression follows a power law with an exponent of 0.6 (Reichenbach and Hudspeth, 2014; Biesmans et al., 2017). We therefore transformed the envelope through this power law before bandpass filtering it.

For the decoding approach (backward models) the envelope was bandpass filtered in either the delta, theta or alpha range (filters as described for the neural data), and downsampled to 50 Hz, analogous to the EEG recordings. For the encoding approach (forward models), and in the noncausal approach, the envelope was gently low-pass filtered [linear phase FIR filters, cutoff (−6 dB) 31 Hz, order 86, transition bandwidth 38 Hz, one-pass forward and compensated for delta], and downsampled to 100 Hz. In the causal approach, the envelope was filtered with an anti-causal minimum-phase low-pass filter [envelope flipped in time and filtered by a causal filter; FIR filter, cutoff (−6 dB) 31 Hz, order 86, transition bandwidth 38 Hz] before downsampling to 100 Hz.

##### Cortical entrainment to the speech envelope.

First, single-lag linear backward models were computed to reconstruct the attended speech envelope from the neural recordings. Separate models were constructed for each subject, each acoustic stimulus and for each frequency band. At each time point *t_n_* the envelope *y* of the target speech was modeled as a linear combination of the EEG channels with a given lag τ*_k_* : ŷ(*t_n_*) = ∑_*j*=1_^*N*^β_*j*,*k*_*x_j_*(*t_n_* + τ*_k_*), where *ŷ* is the reconstructed envelope, *x_j_* denotes the activity at channel *j*, and β*_j,k_* are a set of coefficient to determine. Lags τ*_k_* from −200 to 400 ms in increments of 20 ms were used, resulting in *M* = 31 lags. The models at each lag were estimated using a regularized ridge regression coupled with a fivefold cross-validation (Hastie et al., 2009). The performance of the models was obtained from the testing data by dividing the predicted envelope *ŷ* and the actual envelope *y* into 10-s-long segments and computing the Pearson's correlation coefficient between these segments. For each subject, condition and frequency band, we thus obtained a set of correlation coefficients *c*_*k*_^(*l*)^. The index *k* hereby denotes the lag τ*_k_* used in the backward model, and *l* denotes the testing segment: 1 ≤ *l* ≤ *N*_s_ with *N_s_* the number of 10 s segment in the testing data. The correlation coefficients *c*_*k*_^(*l*)^ quantified how accurately the attended speech envelope could be predicted from the EEG recording at a specific lag.

##### Decoding speech clarity and comprehension.

The correlation coefficients *c*_*k*_^(*l*)^ were used to train two linear models that predicted clarity or comprehension of all 10 s testing segments: *ẑ*(*s_l_*) = ∑_*k*=1_^*M*^ρ*_k_c*_*k*_^(*l*)^, where *k* indexes the lag, *s_l_* denotes a testing segment, *ẑ* is the predicted clarity or comprehension, and ρ*_k_* are the coefficients to determine. This model can be viewed as a backward model that reconstructs a property of the acoustic stimulus from the EEG recordings.

For each subject and each frequency band (delta, theta, alpha), a single model was fitted that reconstructed the clarity for all 10 s testing segments. Speech comprehension was reconstructed likewise through single models for each subject and frequency band. Critically, all eight acoustic stimuli were fitted by the same model, thus ensuring that acoustic cues could not be used when predicting comprehension and that differences in comprehension could not be used for decoding clarity. The models were trained using a regularized ridge regression with fivefold cross-validation, and the regularization parameter that yielded the lowest root-mean-square error (RMSE) on the testing data were selected to yield the best model. To investigate the temporal features of the model we considered its forward version (Haufe et al., 2014).

Two null distributions were established by shuffling the clarity or comprehension values randomly between the different conditions (same set of values but attributed to random conditions), and by computing null backward and forward models that related the shuffled values to the cortical tracking. The shuffling and subsequent model computation was repeated 1000 times. When comparing the coefficients of the forward models to the null distributions, the significance threshold was corrected by FDR over the time lags.

##### Encoding of speech clarity and comprehension.

We modeled the neural activity at each electrode and in each frequency band as the linear superposition of three features. First, we accounted for the acoustic background through the envelope of the babble noise *y_B_*. Second, we considered a feature that reflected the speech clarity by multiplying the envelope of the target speech *y_T_* by the clarity value *a*. Third, comprehension was described through a feature that multiplied the envelope of the target speech *y_T_* by the comprehension value *c*. Multiplying the speech envelope by the clarity as well as by the comprehension of speech thereby yielded two continuous features that encoded the corresponding speech property and that could be related to neural responses through temporal response functions (TRFs). The estimated EEG response ê*_j_* at channel *j* thus follows as ê*_j_* = (1 − *a*) · *T*_*B*_^(*j*)^ * *y_B_* + *T*_*A*_^(*j*)^ * (*a* · *y_T_*) + *T*_*C*_^(*j*)^ * (*c* · *y_T_*), where * is the convolution symbol and where *T*_*B*_^(*j*)^, *T*_*A*_^(*j*)^, and *T*_*C*_^(*j*)^ denote the TRF for the background noise, speech clarity and speech comprehension, respectively.

The TRFs were computed using either the EEG data and the speech envelope both processed by noncausal filters (delta, theta, and alpha), or by using the EEG data and the speech envelope both processed by the causal filters. For each subject, the data from all eight acoustic conditions was pooled together, and the model was fitted using a regularized ridge linear regression. The obtained TRFs were then averaged over all subjects.

To assert the significance of the obtained TRFs, empirical null distributions were established by shuffling the EEG recordings between the acoustic conditions, that is, by mismatching the EEG data and the envelopes, and by computing the resulting TRFs as described in this section. The shuffling was repeated 5000 times. Finally a null distribution was established for each channel, feature, and frequency band by pooling together the corresponding null responses in the −500 to 700 ms time range, and fitting a Gaussian distribution to the resulting data. These distributions were used to compute *p* = 0.01 thresholds (two-tailed with Bonferroni correction for multiple comparison). The TRFs that represented the actual neural responses were then compared with these thresholds.

## Results

### 

#### Behavioral assessment

**Figure 2.**
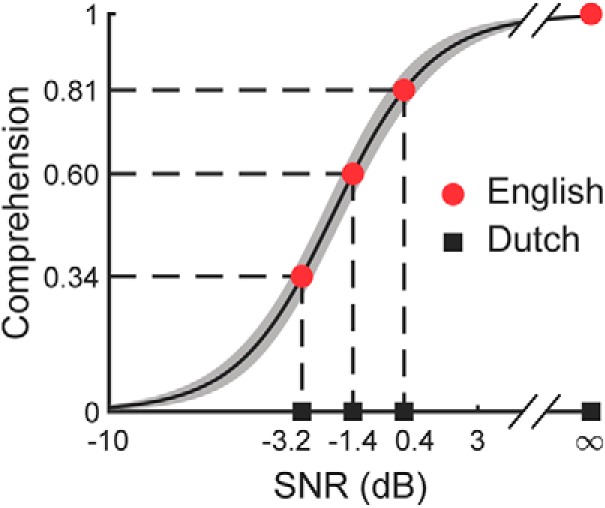
Behavioral responses. The mean comprehension of English in babble noise (black line) increased most from −5 to 2 dB SNR, and had only a small SD across subjects (gray shading). The subsequent EEG recordings used SNRs of −3.2, −1.4, and 0.4 dB as well as no background noise that led to low, medium, high, and full comprehension for the English stimuli (red disks) but yielded no comprehension for the Dutch stimuli (black squares).

We first characterized the comprehension of English sentences in varying levels of background noise behaviorally. We used multi-talker babble noise as a real-world type of background noise (Wilson, 2003), and characterized its level through the signal-to-noise ratio (SNR) of the embedded target speech. We used a speech-in-noise test to quantify the dependence of comprehension on the SNR. The sigmoidal dependence can be described by two parameters: the SNR that yields a comprehension of 50%, and the slope of the sigmoidal curve at that point (Fig. 2). We obtained a SNR of −2.0 dB at 50% comprehension, and a slope of 0.15 dB^−1^. From the resulting sigmoidal curve we could infer that the comprehension at a SNR of −3.2 dB was 34%, that the comprehension at a SNR of −1.4 dB was 60%, and that the comprehension at a SNR of 0.4 dB reached 81% (Fig. 2). We refer to these three SNRs as high, medium, and low babble noise in the following (Table 1). Moreover, we found speech comprehension to be consistent among the different individuals: the SD across participants was only 5.8% at the SNR of −2.0 dB that produced 50% comprehension, and was smaller at both lower and higher SNRs. For the subsequent EEG recordings we therefore used English as well as Dutch speech in the low, medium, and high babble noise, as well as in quiet.

#### Cortical tracking of the speech envelope

As a first step toward the decoding of speech clarity and comprehension from the EEG recordings, we quantified the strength of the cortical entrainment to the target speech envelope at different time lags and in the delta, theta, and alpha frequency bands separately. In particular, we reconstructed the attended envelope from the EEG recordings through a linear backward model. The correlation of the reconstructed envelope to the speech envelope informed on the magnitude of the cortical entrainment.

**Figure 3.**
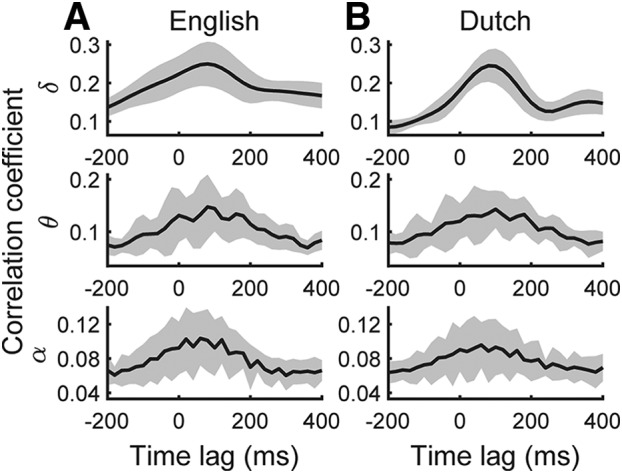
Population average (black) of the cortical tracking of the speech envelope in quiet, and its SD across subjects (gray). ***A***, Neural entrainment to the English speech material. The cortical tracking in the delta band and theta band was strongest at a delay of 80 ms, whereas the alpha band tracked the envelope most at a latency of 60 ms. ***B***, The neural tracking of the Dutch speech showed peaks at the same delays as for the responses to English. The neural tracking of the Dutch speech showed peaks at similar latencies as for the responses to English. Moreover, at the latency of peak average entrainment, the amplitude did not differ significantly between English and Dutch.

We found that, for English presented without background noise, the cortical tracking in the delta and theta band was strongest at time lags of 80 ms (Fig. 3*A*). Entrainment of the alpha-band activity peaked at 60 ms, and was the weakest, presumably reflecting weaker phase locking to the envelope at higher frequencies. For each frequency band, the temporal spread around the peak was on the order of a few hundred milliseconds, corresponding to the inverse of the width of the frequency band.

**Figure 4.**
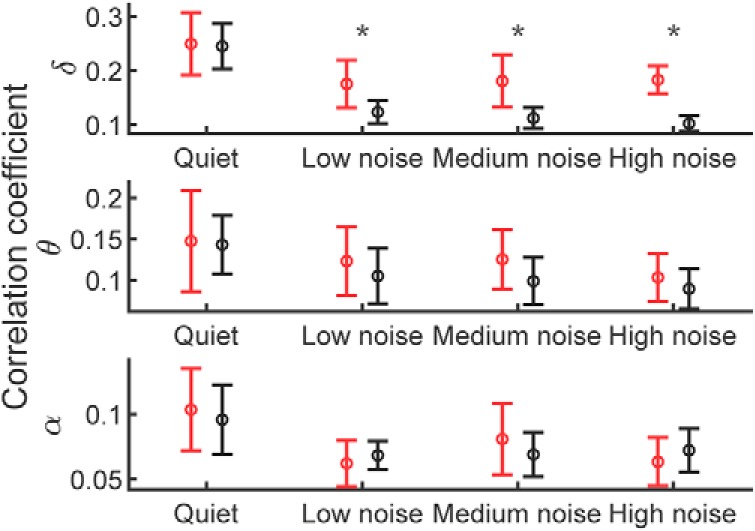
Amplitude of the cortical tracking at peak average entrainment to the speech envelope for English and Dutch in different levels of background noise. The neural entrainment at the peak latency was comparable between English and Dutch in both the theta and the alpha frequency bands. However, the cortical tracking of English was significantly higher than that of Dutch in the delta band in the presence of background noise. *p = 0.001.

The cortical tracking of the Dutch audio stimuli, without background noise, was very similar to that of the English speech material (Fig. 3*B*). In particular, the tracking of the Dutch speech envelope in the different frequency bands did not peak at a different latency as for the English speech envelope (*p* > 0.05, two-tailed paired Wilcoxon signed rank test). Moreover, at the latency of peak average entrainment, the amplitude did not differ significantly between the Dutch and the English speech material (*p* > 0.05, two-tailed paired Wilcoxon signed rank test). When comparing the cortical tracking of speech in different levels of background noise across languages, the amplitude at peak average entrainment of the neural response only differed significantly between English and Dutch in the delta band (Fig. 4; *p* = 0.001, two-tailed paired Wilcoxon signed rank test, FDR correction for multiple comparison), but not in the theta or alpha band. This suggested that speech comprehension might be reflected by cortical tracking in the delta band.

#### Decoding of speech clarity and comprehension from EEG

**Figure 5.**
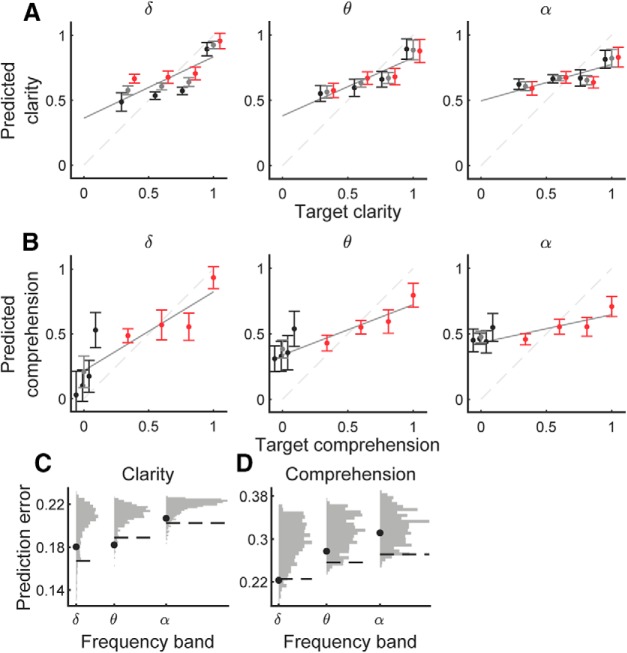
Predictions of speech clarity as well as comprehension. Clarity (***A***) and comprehension (***B***) were predicted from the neural tracking of the speech envelope in different frequency bands (disks, population mean; error bar, population SD; black, Dutch; red, English; gray, mean. Note that the points were jittered along the *x*-axis so they could be visually distinguished). ***C***, ***D***, the null distributions (gray) allow to compute the threshold of the RMSE (dashed line) below which the prediction of speech clarity or speech comprehension (black disks) is significant (*p* < 0.05). ***C***, The prediction of clarity is significant for the theta band. ***D***, Only the delta band allows significant decoding of speech comprehension.

We computed two linear models, one model to predict speech clarity and another to estimate comprehension, from the strength of the cortical entrainment to the speech envelope in the delta, theta, and alpha band and at different delays. We found that the cortical tracking in the different frequency bands and at the various lags allowed to estimate both speech clarity as well as comprehension (Fig. 5*A*,*B*). When predicting speech clarity the model predicted comparable values for English and for Dutch at the same SNR. Similarly, when predicting comprehension, the predictions for the Dutch stimuli in the different noise levels were all similar. The predictions of speech clarity and comprehension therefore followed the same trend regardless of the language and were not driven by a particular category of stimuli.

To ascertain the statistical significance of the predictions of speech clarity and comprehension, we compared the results to empirical null distributions. The null distributions were obtained by shuffling the clarity or comprehension values at random between the different acoustic conditions. The null models then attempted to estimate the randomized clarity or comprehension values from the cortical entrainment. We quantified the performance of the null models through their RMSE, and used the distribution of the RMSEs to determine whether the RMSE of the sensical models were significant (Fig. 5*C*,*D*).

We found that the cortical tracking in the theta band allowed a statistically-significant prediction of clarity (*p* = 0.017; Fig. 5*C*). The RMSE of the clarity prediction was not significant when using the alpha band (*p* = 0.080), or the delta band (*p* = 0.14).

Conversely, the best decoding of speech comprehension was achieved from the cortical tracking in the delta band, which was also statistically significant (*p* = 0.038; Fig. 5*D*). Prediction of speech comprehension was not significant when based on the theta band (*p* = 0.22) or the alpha band (*p* = 0.45).

#### Time lags of neural speech tracking that inform on clarity and comprehension

**Figure 6.**
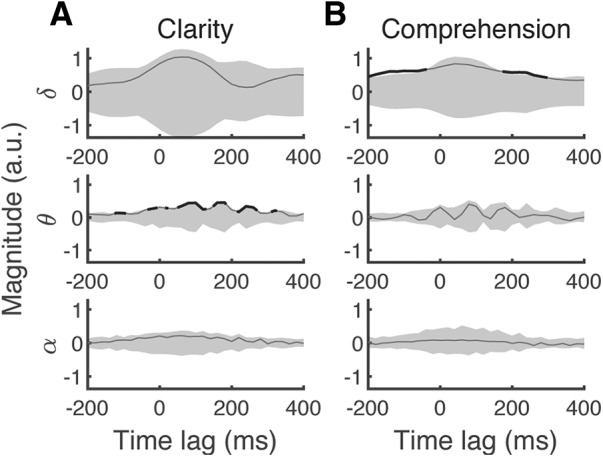
Contribution of the cortical speech tracking at different latencies to speech clarity and comprehension. The population averages (black lines) of the coefficients of the forward model predicting neural entrainment from clarity (***A***) or from comprehension (***B***) are compared with the null distribution obtained by shuffling the clarity respectively comprehension values (gray shading, two-tailed, *p* = 0.05, FDR corrected over time lags). Latencies at which the coefficients of the forward model reach statistical significance are indicated through a bold line. ***A***, Clarity relates to the neural entrainment in theta band at a delay of 160 ms. ***B***, Comprehension modulates delta band entrainment at −100 and 240 ms.

We wondered which temporal lags of the cortical speech tracking in the different frequency bands informed most on speech clarity and comprehension. We accordingly computed two linear forward models that reconstructed the strength of the neural entrainment at the different delays and in the different frequency bands from the speech clarity and the speech comprehension. The coefficients of these two models revealed how strongly each feature, that is, the strength of the cortical entrainment to the speech envelope at each time lag and in each frequency band, correlated with speech clarity and comprehension (Fig. 6). We found that the theta entrainment at 160 ms correlated most with speech clarity. For speech comprehension, however, the delta entrainment at a latency of −100 and 240 ms was most informative. The entrainment at the −100 ms delay hereby preceded the acoustic signal.

#### Encoding of speech clarity and comprehension in the neural response

We sought to confirm the results from our decoding approach by running a complementary encoding analysis. To this end, we determined how speech clarity and comprehension are encoded in the EEG response through computing a forward model. In particular, the neural response in each frequency band and at each electrode was estimated from three features of the audio stimuli. The first feature was the envelope of the babble noise and accounted for the acoustic background. The second and third features were the speech envelope weighted by the speech clarity and the speech comprehension, respectively (see Materials and Methods). The estimation of the EEG data from these three features resulted in three TRFs. A null distribution for the TRFs was computed as well by mismatching the EEG recordings and the acoustic features.

**Figure 7.**
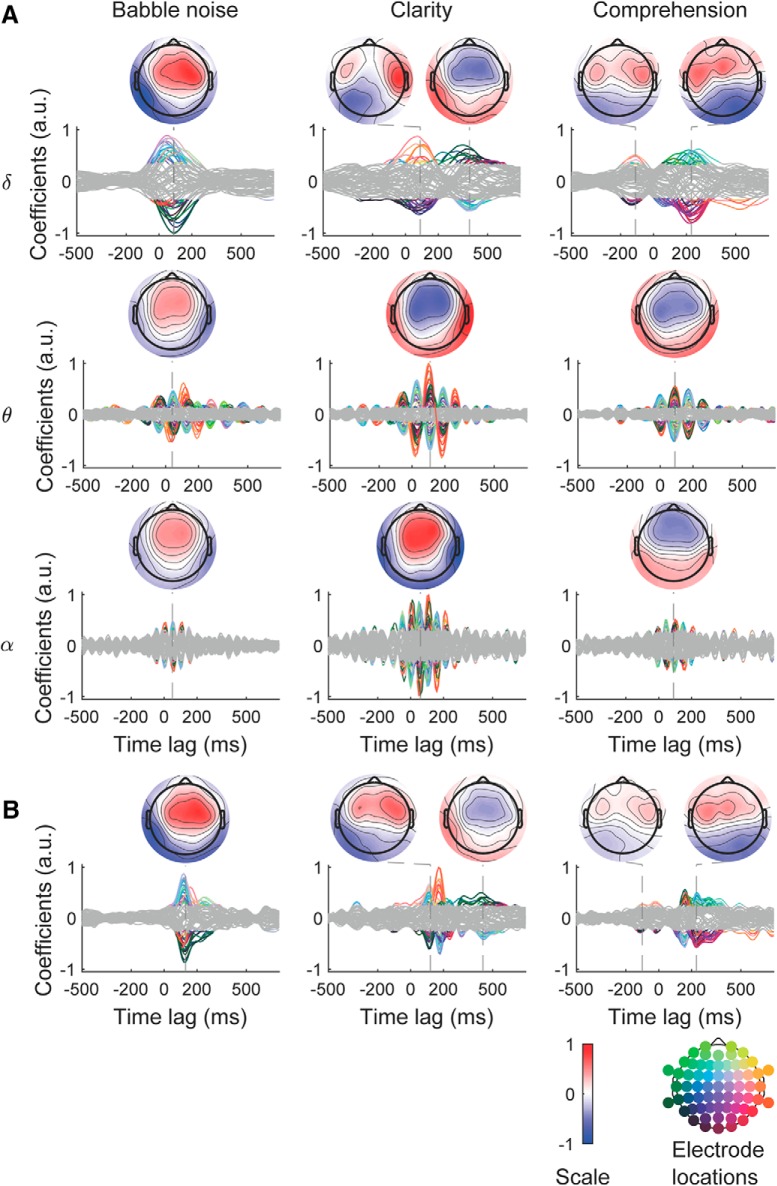
Neural encoding of speech clarity and comprehension. ***A***, TRFs obtained from noncausal filtering describing the responses to the babble noise (left), clarity (center), and comprehension (right) at each electrodes and in different frequency bands. The significance threshold is indicated in gray. Both the background noise and speech clarity are encoded at positive lags, reflecting the lower-level acoustic nature of these features. In contrast, speech comprehension is partly encoded at a negative lag of −100 ms in the delta band, hinting at an early component of speech comprehension. ***B***, We also computed TRFs using causal filters. These TRFs capture both delta and theta band activity, and are strikingly similar to those obtained from the noncausal filters. However, their timing exceeds the one of the TRFs computed from noncausal filters by 50 ms.

All TRFs exhibited regions where they were significantly different from the null distributions (Fig. 7*A*; *p* < 10^−2^ mass univariate two-tailed test with Bonferroni correction for multiple comparison). In the delta band, the responses to the noise were found to exhibit a right lateralized peak at 80 ms, and no later components. In contrast, the neural response to speech clarity exhibited two distinct peaks. The first one was right lateralized and at a delay of 90 ms, whereas the second one was left lateralized and at a latency of 390 ms. The EEG response to speech comprehension had significant contributions at both negative and positive latencies. The lateralization changed from left at a latency of −110 ms to right at 230 ms, with a polarity inversion at 100 ms. In the theta band, the neural responses were symmetrical between the hemispheres and peaked at a latency of 40, 110, and 90 ms for the noise, clarity, and comprehension, respectively. In the alpha band, we observed weak and noisy responses presumably reflecting the weaker phase locking of EEG neural data to the speech envelope at higher frequencies. They peaked at 55 ms for the background noise and speech clarity, and at 90 ms for comprehension.

To investigate the role of the filters in the shape of the TRFs, and in particular their role in the negative latency of −100 ms that emerged for speech comprehension, we used causal filters as well. Causal filters will strictly preserve the causal relationship between input and output, but typically introduce phase distortions. We found that causal filters resulted in strikingly similar TRFs to the ones we obtained for the delta band from the noncausal filters (Fig. 7*B*). In particular, the early component in the TRFs for speech comprehension was conserved.

## Discussion

Using natural speech in a subject's native as well as in a foreign language, and in different levels of babble noise, allowed us to separate neural correlates of speech clarity from those of speech comprehension. We used this experimental paradigm to disambiguate the role of the cortical entrainment to the speech envelope for the processing of lower-level acoustic features as well as of higher-level linguistic ones.

We found that only cortical speech tracking in the theta frequency band could significantly predict speech clarity, reflecting the stimulus acoustics, whereas delta band tracking alone yielded a significant prediction of speech comprehension, associated to higher-level linguistic processing. Although we considered clarity and comprehension as independent, there likely are interactions between the two variables that our model could not capture. Our finding that the cortical speech tracking in the delta band relates most to speech comprehension agrees with recent results on the delta band for linguistic processing (Kösem and Van Wassenhove, 2017). In particular, cortical activity in the delta band has been found to track syntactic structure in speech as well as the semantic dissimilarity between successive words (Ding et al., 2016; Broderick et al., 2018). Both syntactic as well as semantic aspects of speech represent high-level linguistic features that reflect speech comprehension and that cannot be perceived in a foreign language. Moreover, the mutual information between neural activity in the delta band and the speech envelope has been shown to be larger when subjects correctly understood speech in noise than when they misunderstood the target speech (Keitel et al., 2018), and delta-band neural coherence was stronger when subjects listened to speech compared with amplitude-modulate noise or spectrally-rotated speech (Molinaro and Lizarazu, 2018). These magnetoencephalographic studies allowed to spatially localize the neural sources to the auditory cortex and more frontal regions, in particular the premotor cortex. The EEG measurements that we have analyzed here showed scalp topographies that were consistent with these findings, evidencing neural origins in the temporal areas, in particular from the auditory cortices and associated areas (Fig. 7).

Our results highlight in particular that delta band entrainment at 100 ms before the stimulus is significantly related to comprehension. We have shown that causal filters whose output does not depend on future inputs leads to a similar early neural component, which therefore does not appear to be an artifact of a particular filter design or acausal filtering. This preceding component could indicate a predictive mechanism involved in speech comprehension. Predictive processing such as through probabilistic word pre-activation has indeed been implicated in spoken language comprehension (DeLong et al., 2005; Otten et al., 2007; Friston and Kiebel, 2009; Kutas et al., 2011; Lewis and Bastiaansen, 2015). A predictive neural component is also consistent with a recent report that envelope-shaped transcranial current stimulation preceding aural input improves speech comprehension (Riecke et al., 2018).

Alternatively, the preceding neural component may reflect neural activity that originates after the acoustic stimulus, but that is masked by other neural activity at the positive lags. Indeed, the spectral filtering of the EEG response into the different frequency bands introduces a smearing of the timing of the response. A neural signal related to speech comprehension at an early positive delay will accordingly show contributions at somewhat shorter and longer delays as well, including at negative latencies if noncausal filters are used (Fig. 3*A*). If another later neural component of cortical entrainment exists that is not related to speech comprehension and whose temporal range partially overlaps with that of the first neural component, then only the earliest lags associated to the first component that do not overlap with the second later component may be predominantly associated to speech comprehension. Such an early neural component for speech comprehension is consistent with a recent finding on a rapid transformation from auditory to linguistic speech structures (Brodbeck et al., 2018).

The role of the theta band for the lower-level acoustic processing that we have identified may reflect the role of these faster neural activities in the tracking of shorter acoustic features such as syllables and phonemes (Di Liberto et al., 2015; Brodbeck et al., 2018). Although we found significant entrainment to the speech envelope in the alpha band, this did not contribute significantly to the decoding of either speech clarity and speech comprehension. Although neural activity in the alpha band has previously been found to correlate to aspects of speech processing, this contribution emerged indeed for the power in the alpha band but not for its entrainment to the speech envelope (Obleser and Weisz, 2012; Strauβ et al., 2014a,b; Kayser et al., 2015).

Although we assumed a monotic relationship between the cortical tracking of the speech envelope and speech clarity as well as comprehension, some of our results suggest that this may not be the case at relatively high levels of the SNR, ∼0.4 dB, as is apparent in the relation between predicted to target speech clarity (Fig. 5). Future studies that use a larger number of SNRs, particularly at higher values, may further clarify this issue.

Beyond evidencing different functional roles of the cortical entrainment in the delta and theta frequency bands, our results also show how speech intelligibility and speech comprehension can be decoded from short segments of EEG recordings of a duration of 10 s only. The decoding of speech comprehension may be particularly useful for future auditory brain–computer interfaces (BCIs). As an example, an auditory BCI may allow a locked-in patient with traumatic brain injury who cannot communicate overtly to be assessed for their speech comprehension and consciousness (Laureys et al., 2004; Goldfine et al., 2011). Moreover, an auditory BCI to decode speech comprehension may guide the settings in an auditory prosthesis, such as a hearing aid or a cochlear implant, to adapt if the wearer's comprehension drops because of changes in the acoustic environment, which may enable the wearer to better understand speech in different types of background noise (Knudsen et al., 2010).

Practical applications of the decoding of speech comprehension will benefit from further optimization of the decoding. In particular, we have used linear models because of their interpretability and robustness. However, speech clarity and comprehension exhibit a sigmoidal dependence on the SNR, and adding a corresponding sigmoidal function in the model may improve the prediction. Additionally this procedure could be refined by exploiting the spatial patterns highlighted by our encoding analysis. Moreover, nonlinear models such as deep neural networks are able to extract highly nonlinear dependencies from the input data including EEG recordings, which may improve decoding even further (Sturm et al., 2016; Schirrmeister et al., 2017). Our work opens the door to such further investigations that will increasingly clarify the different intertwined neural mechanisms of speech processing as well as use them for technological applications.
